# Dose-Dependent Effects of Lipopolysaccharide on the Endothelium—Sepsis versus Metabolic Endotoxemia-Induced Cellular Senescence

**DOI:** 10.3390/antiox13040443

**Published:** 2024-04-09

**Authors:** Dennis Merk, Fiona Frederike Cox, Philipp Jakobs, Simone Prömel, Joachim Altschmied, Judith Haendeler

**Affiliations:** 1 Environmentally-Induced Cardiovascular Degeneration, Clinical Chemistry and Laboratory Diagnostics, Medical Faculty, University Hospital and Heinrich-Heine-University, 40225 Düsseldorf, Germany; dennis.merk@hhu.de (D.M.); fiona.cox@hhu.de (F.F.C.); philipp.jakobs@hhu.de (P.J.); 2 Medical Faculty, Institute for Translational Pharmacology, University Hospital and Heinrich-Heine-University, 40225 Düsseldorf, Germany; 3 Department of Biology, Institute of Cell Biology, Heinrich-Heine-University, 40225 Düsseldorf, Germany; proemel@hhu.de; 4 Medical Faculty, Cardiovascular Research Institute Düsseldorf, CARID, University Hospital and Heinrich-Heine-University Düsseldorf, 40225 Düsseldorf, Germany

**Keywords:** endothelial cells, high-dose endotoxemia, lipopolysaccharide, low-dose endotoxemia, stress-induced endothelial cell senescence

## Abstract

The endothelium, the innermost cell layer of blood vessels, is not only a physical barrier between the bloodstream and the surrounding tissues but has also essential functions in vascular homeostasis. Therefore, it is not surprising that endothelial dysfunction is associated with most cardiovascular diseases. The functionality of the endothelium is compromised by endotoxemia, the presence of bacterial endotoxins in the bloodstream with the main endotoxin lipopolysaccharide (LPS). Therefore, this review will focus on the effects of LPS on the endothelium. Depending on the LPS concentration, the outcomes are either sepsis or, at lower concentrations, so-called low-dose or metabolic endotoxemia. Sepsis, a life-threatening condition evoked by hyperactivation of the immune response, includes breakdown of the endothelial barrier resulting in failure of multiple organs. A deeper understanding of the underlying mechanisms in the endothelium might help pave the way to new therapeutic options in sepsis treatment to prevent endothelial leakage and fatal septic shock. Low-dose endotoxemia or metabolic endotoxemia results in chronic inflammation leading to endothelial cell senescence, which entails endothelial dysfunction and thus plays a critical role in cardiovascular diseases. The identification of compounds counteracting senescence induction in endothelial cells might therefore help in delaying the onset or progression of age-related pathologies. Interestingly, two natural plant-derived substances, caffeine and curcumin, have shown potential in preventing endothelial cell senescence.

## 1. Cardiovascular Diseases and Endothelial Dysfunction

Cardiovascular diseases (CVDs) are the leading cause of morbidity and mortality in the world and were responsible for 17.9 million deaths in 2019 (https://www.who.int/news-room/fact-sheets/detail/cardiovascular-diseases-(cvds) (accessed on 7 February 2024). Their development and progression are intimately linked to endothelial dysfunction caused by factors such as oxidative stress. The vascular endothelium consists of a monolayer of endothelial cells, which constitutes the inner lining of all blood vessels. Endothelial cells are attached to the basal lamina, which acts as a scaffold, with pericytes and smooth muscle cells situated on the outside. The endothelium thereby forms a barrier between the bloodstream and the surrounding tissue and maintains vascular homeostasis. It secretes multiple factors that influence coagulation, vascular tone, adhesion of leukocytes, and inflammation, underscoring the importance of the endothelium for vascular homeostasis [1]. Endothelial cells are also important for the exchange of nutrients and molecules between the blood and the surrounding tissue. Their functionality requires energy to maintain a healthy endothelium [2].

Endothelial dysfunction is defined as the failure of endothelial cells to carry out their normal functions and is a consequence of their excessive activation and apoptosis [3]. The activation is induced by proinflammatory cytokines, including Tumor Necrosis Factor (TNF)-α and Interleukin-6 (IL-6) [4]. It is characterized by the expression of cell-surface adhesion molecules, such as Intercellular Adhesion Molecule 1 (ICAM1) or Vascular Cell Adhesion Molecule 1 (VCAM1). These adhesion molecules facilitate the recruitment and attachment of leukocytes to the endothelium, leading to increased endothelial leakiness, allowing diapedesis—the transmigration of leukocytes through the endothelial cell layer [3]. Prolonged endothelial cell activation and the unrestricted secretion of inflammatory cytokines by leukocytes favor endothelial cell apoptosis. Apoptosis is executed by caspases, which cleave proteins critical for cell survival [5]. Tight control of apoptosis is a prerequisite for maintaining a healthy endothelium, especially because endothelial cells are the first cells exposed to invading pathogens. Their dysfunction is a precursor to tissue and organ damage and is a major contributor to certain lethal pathologies, e.g., sepsis. Thus, the next two chapters will introduce the mechanisms in sepsis/high-dose endotoxemia with a specific focus on the endothelium.

## 2. Sepsis

**Figure 1:**
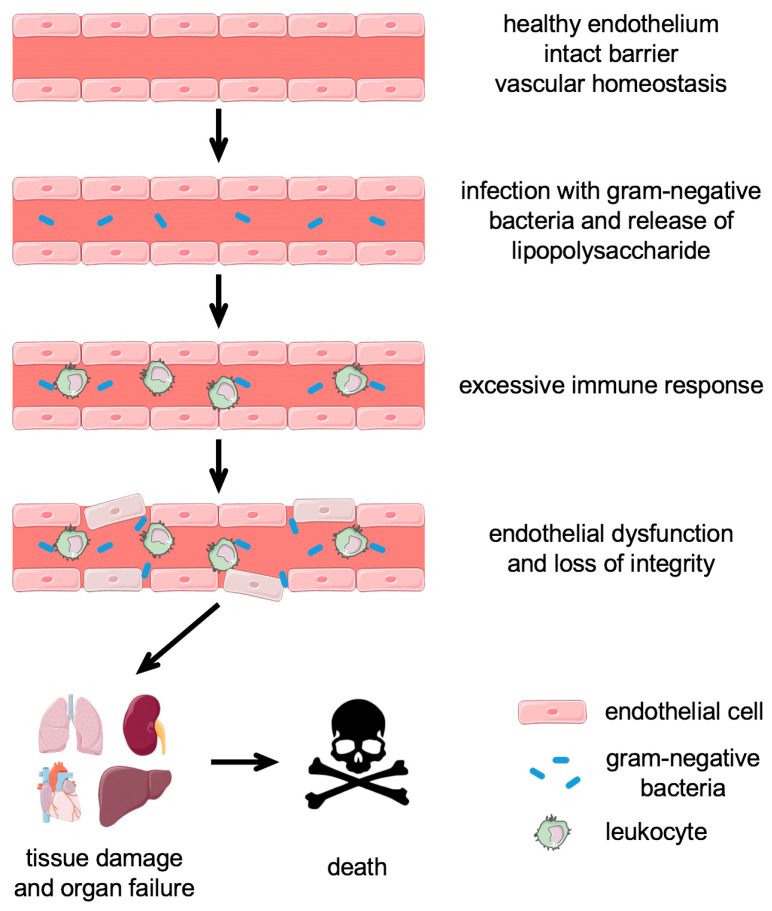
Sepsis progression. Under physiological conditions, the endothelium is an intact barrier between the bloodstream and surrounding tissues and maintains vascular homeostasis. Upon systemic infection with gram-negative bacteria, lipopolysaccharide is released, leading to the activation of endothelial cells. The excessive secretion of cytokines by recruited leukocytes leads to the breakdown of the endothelial barrier. This results in tissue damage and organ failure and can ultimately lead to death.

Sepsis and septic shock are the leading cause of mortality in critically ill patients, accounting for approximately 20% of all-cause deaths globally. It was estimated that sepsis had affected 49 million individuals and was related to 11 million deaths worldwide in 2017 [6]. Sepsis is defined as a life-threatening organ dysfunction caused by a dysregulated host response to infection [7]. Its pathogenesis includes various steps, such as infection, hyperactivation of the immune system, endothelial dysfunction leading to loss of barrier integrity, followed by tissue damage, and finally, multi-organ failure and death [8] (Figure 1).

Infection with gram-negative bacteria, including *Escherichia coli* and *Pseudomonas aeruginosa*, is the prevailing cause of severe sepsis in humans [9]. Under normal circumstances, the innate immune system reacts to local infections and protects the organism from further damage. However, the hyperactivity of the immune response in sepsis occurs when the host loses control of the local containment of an infection, such that it becomes systemic. This leads to the production of proinflammatory cytokines, reactive oxygen species (ROS), and cellular injury, mediated by the recognition of pathogens by pattern recognition receptors (PRRs) [10,11]. Those PRRs, which are expressed on the surface of immune and phagocytic cells, and also on some non-immune cells, recognize pathogen-associated molecular patterns (PAMPs) that are expressed on both harmless and pathogenic microbes [11]. During tissue damage, intracellular components are released, which are recognized as damage-associated molecular patterns (DAMPs), and activate the immune system in concert with PAMPs [12].

The most important PAMP in the context of sepsis is lipopolysaccharide (LPS), an outer membrane component of gram-negative bacteria. LPS consists of a proximal oligosaccharide core, the distal O antigen, a glycan polymer, and a lipid A hydrophobic anchor—a glucosamine disaccharide decorated with multiple fatty acids [13]. Its immunostimulatory activity is mostly derived from the lipid A moiety, which in humans and mice binds to toll-like receptors (TLRs) and thereby activates the innate immune response [14]. LPS can exert its effect either locally or, after entering the blood circulation, systemically. LPS also affects the endothelial cell transcriptome by regulating transcripts of protein coding and non-coding RNAs [15]. Endothelial cells treated with LPS showed a significant increase in upregulated genes with gene ontology (GO) terms related to immune response to bacteria and TNF signaling [16]. Moreover, LPS concentrations above approximately 500 pg/mL are known to be lethal. This was shown in a study with 253 patients at the onset of severe sepsis and/or septic shock, where the median endotoxin concentration in the plasma was 300 pg/mL. More importantly, in survivors over a 28-day period, the mean level at study entry was 230 pg/mL, while it was 515 pg/mL in non-survivors [17].

Although TLR signaling is well described (see chapter 3), LPS effects are more complex and multifaceted, and the underlying mechanisms are far from understood. This explains why there are still no FDA-approved treatment options available that improve sepsis survival, despite significant advancements in therapeutic management and over 100 therapeutic trials [18,19]. Further exploration of the endothelial cell response to LPS and possible interference with the negative effects might provide clues for new targets for adjuvant therapies aimed at protecting the endothelium against damage and preventing the breakdown of the endothelial barrier.

## 3. Toll-like Receptors

**Figure 2:**
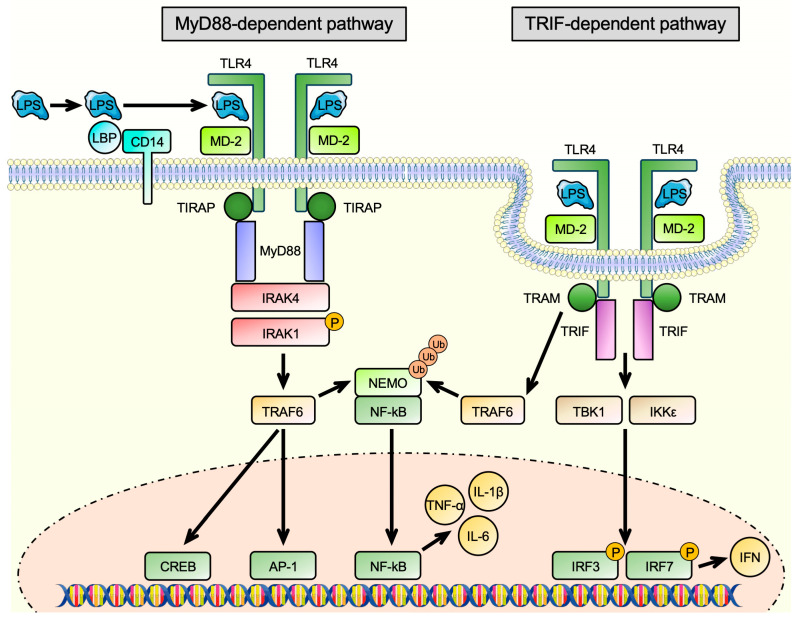
Lipopolysaccharide-induced Toll-like Receptor 4 signaling. After entering the circulation, lipopolysaccharide (LPS) is bound by Lipopolysaccharide-Binding Protein (LBP) and—facilitated by CD14—transferred to Myeloid Differentiation Protein 2 (MD-2). Together, LPS and MD-2 form a complex with Toll-like Receptor 4 (TLR4), which homodimerizes. This triggers two distinct, consecutive pathways: (1) the MyD88-dependent pathway and the (2) TRIF-dependent pathway. First, TLR4 interacts with the TIR Domain Containing Adapter Protein (TIRAP), which recruits MyD88. Following its association with the complex, IL-1 Receptor Associated Kinase 4 (IRAK4) phosphorylates IRAK1, which in turn couples to TNF Receptor Associated Factor 6 (TRAF6). TRAF6 activates the transcription factors cAMP Response Element Binding Protein (CREB) and Activator Protein-1 (AP-1) and promotes polyubiquitination of NEMO, an inhibitor of nuclear factor-κB (NF-κB) nuclear translocation. Its degradation allows nuclear entry of NF-κB and the expression of pro-inflammatory cytokines, such as IL-6, IL-1β, and TNF-α. In the second step, TLR4 dissociates from TIRAP and MyD88 and undergoes endocytosis. Now, the TRIF-Related Adapter Molecule (TRAM) enables binding of the TIR Domain Containing Adaptor Molecule 1 (TRIF). TRIF-dependent signaling activates two kinases, TANK Binding Kinase 1 (TBK1) and IκB Kinase ε (IKKε), which phosphorylate the transcription factors Interferon Regulatory Factor (IRF) 3 and 7, leading to the expression of type I interferons (IFN), enhancing the inflammatory response even further. In addition, TRAM also interacts with TRAF6, supporting NF-κB activation through the degradation of NEMO.

TLRs are a family of transmembrane glycoproteins, which, in humans, is comprised of 10 members. They are located on macrophages, T-cells, B-cells, dendritic cells, and also non-immune cells, like endothelial cells [20,21]. They recognize and bind to a wide range of different molecules, including microbial components, such as LPS, lipopeptides, or double-stranded RNA, via a horseshoe-shaped ligand binding domain built from leucine-rich repeats [22]. The binding of ligands induces a rearrangement of the receptor complex and induces the innate immune response and inflammation [14]. They detect different microbial components, such as LPS, lipopeptides, or double-stranded RNA, and induce the innate immune response and inflammation [14]. The most important TLR in LPS signaling is TLR4 [23]. According to the publicly available databases Genecards [24], Human Protein Atlas [25], and GTEx Portal [26], its transcript is present in nearly all human tissues. TLR4 can not only be found on blood cells but also on the cell surface of endothelial cells [27], smooth muscle cells [28], skeletal muscle cells [29], cardiac myocytes [30], and cells of the central nervous system [31]. The lipid A moiety of LPS is recognized and first bound by lipopolysaccharide-binding protein (LBP) and transferred to CD14 molecules. Membrane-bound CD14 (mCD14) is a glycosylphosphatidylinositol (GPI)-anchored protein located on the surface of myeloid–lineage cells like monocytes, macrophages, and dendritic cells [32]. After binding to CD14, LPS is subsequently transferred to Myeloid Differentiation Protein 2 (MD-2), and this transfer is catalyzed by CD14 [33]. MD-2 is essential for the activation of TLR4 by LPS, which was shown by the lack of response of macrophages derived from MD-2 knockout mice after LPS treatment [34]. In addition to mCD14, a soluble form of CD14 (sCD14), which lacks the GPI anchor, exists, which is also released from non-myeloid cells, including hepatocytes, adipocytes, and intestinal epithelial cells [35]. It is produced by shedding or secretion and is found in serum of normal individuals [36]. It has long been known that sCD14 can make CD14-negative cells like epithelial and endothelial cells responsive to LPS stimulation [37,38]. This is achieved by the high-affinity binding of LPS to sCD14 and the transport of this complex through the bloodstream. Soluble CD14 can then transfer LPS to MD-2 as efficiently as mCD14 [33], such that this reaction can also take place on cells devoid of CD14 themselves [39,40]. At this step, a complex of TLR4/MD2/LPS is formed, which builds a homodimer and triggers two consecutive pathways: (1) The MyD88-dependent pathway, which is induced in the plasma membrane, and (2) the TRIF-dependent pathway, which starts in early endosomes after the endocytosis of the receptor complex [41] (Figure 2).

During the MyD88-dependent pathway, the membrane-anchored TLR4 receptor interacts with the TIR domain-containing adapter protein (TIRAP) and recruits MyD88, triggering a signaling cascade. This cascade includes the recruitment of IL-1 Receptor Associated Kinases (IRAKs) 1 and 4. IRAK4 was shown to phosphorylate and thereby activates IRAK1 upon stimulation [42]. Following phosphorylation, IRAK1 associates with TNF Receptor Associated Factor 6 (TRAF6), which is a mediator of cytokine signaling and stress response pathways [43]. These include the activation of the transcription factors cAMP Response Element Binding Protein (CREB) and Activator Protein-1 (AP-1) [44,45,46]. TRAF6 also promotes the polyubiquitination of NEMO, an inhibitor of nuclear factor-κB (NF-κB) nuclear translocation. This triggers the degradation of NEMO, allowing nuclear entry of NF-κB, which then initiates the production of pro-inflammatory mediators, such as IL-6, IL-1β, and TNF-α, leading to an even further enhanced inflammatory response [41]. Following the dissociation of membrane-bound TLR4 from TIRAP and MyD88, TLR4 undergoes endocytosis and binds TIR Domain Containing Adaptor Molecule 1 (TRIF) and TRIF-Related Adapter Molecule (TRAM). It was shown that TRAM recruits TRIF to the plasma membrane and acts as a bridging adaptor, enabling the interaction of TRIF with TLR4 [47]. LPS promotes the complex building of TLR4 and TRAM and their following translocation into the endosome [48]. The LPS-induced internalization of TLR4 is controlled by CD14, whose importance is highlighted by the increased localization of TRAM to regions enriched with CD14 [49]. Endocytosis terminates the MyD88-dependent signaling, and endosome maturation as well as lysosomal degradation of TLR4 define the extent of the TRIF-dependent signaling [50]. TRIF-dependent signaling activates two kinases, TANK Binding Kinase 1 (TBK1) and IκB Kinase ε (IKKε), which phosphorylate the transcription factors Interferon Regulatory Factor (IRF) 3 and 7, leading to the expression of type I interferons, enhancing the inflammatory response even further [41]. In addition, TRAM also interacts with TRAF6, supporting NF-κB activation through the degradation of NEMO [51].

However, it has to be mentioned that the LPS signal cannot only be transmitted via TLR4. An intracellular inflammasome complex, which acts as a molecular signaling platform in the cytoplasm, has been found to recognize LPS intracellularly and activate a human caspase-4/murine caspase-11-dependent pathway that leads to increase in IL-1β and IL-18 and is sufficient to induce pyroptosis [52,53,54]. TLR4 is dispensable for this response, as demonstrated in TLR4-deficient mice, which were primed with a TLR3 agonist to activate caspase 11 and were as susceptible to LPS-triggered sepsis as wildtype animals [52]. After these initial studies, which used LPS-loaded macrophages, it was later demonstrated that outer membrane vesicles produced by gram-negative bacteria can deliver LPS into the cytosol and trigger caspase-11-dependent effector responses in vitro and in vivo [55]. 

Additionally, it was shown that the shape of LPS can vary, activating different TLRs; for example, TLR2 [56]. This is highlighted by the alternative formation of specific TLR receptor clusters in response to different bacterial products [57]. Moreover, the potency of LPS varies, with the hexa-acylated and phosphorylated LPS of *Escherichia coli* being one of the most potent species, whereas under-acylated, dephosphorylated LPS species have weaker pro-inflammatory activity [58]. This also contributes to the aforementioned structural diversity among LPS and influences its functionality [59]. 

Since TLR4 is the dominant PRR in LPS signaling, it seems reasonable to inhibit TLR-4. However, Eritoran, an analog of lipid A and TLR4 antagonist, which inhibits LPS-triggered TLR4 signaling [60], did not result in reduced 28-day mortality in a phase III clinical trial [17].

Septic patients also exhibit overwhelming oxidative stress, which results from the uncontrolled production of reactive oxygen and nitrogen species from activated immune cells. This explains why antioxidant therapies, using vitamin C, selenium, or N-acetylcysteine, were investigated in sepsis models [61]. However, they did not show any positive effects in humans [11]. 

In contrast to the acute effects of high-dose LPS on the vasculature, the effects of low-dose LPS are usually chronic and related to stress-induced senescence and aging, with aging being a major risk factor for the development of CVDs. In the following chapters of the review, we focus on endothelial senescence, its induction by low-dose endotoxemia, the role of the gut microbiome, and highlight the potential of natural dietary compounds to counteract senescence induction.

## 4. Stress-Induced Senescence in the Endothelium

Stress-induced cellular senescence is a phenomenon that occurs in response to various stressors including oxidative stress, xenobiotics [62], or low-dose endotoxemia [63] and goes along with increased intracellular ROS production. It can have detrimental effects on tissue function and homeostasis, and thus, it plays a significant role in age-related dysfunction of the vascular system and the development of cardiovascular diseases. Cells undergoing senescence sometimes exhibit a senescence-associated secretory phenotype (SASP) [64,65]. The SASP-associated inflammatory molecules expressed by vascular cells in atherosclerotic lesions and LPS-induced cellular senescence exaggerate the pathogenesis of CVDs [66]. Understanding the molecular mechanisms underlying stress-induced senescence may provide valuable insights for the development of therapeutic strategies aimed at ameliorating age-related pathologies.

**Figure 3:**
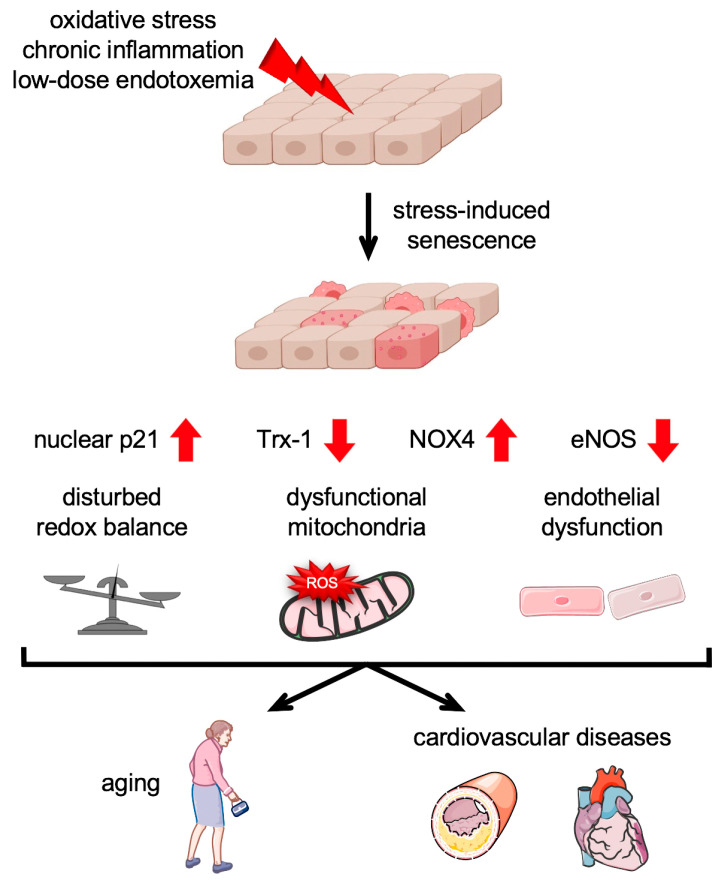
Stress-induced endothelial cell senescence. Endothelial cell senescence is triggered by various stressors including oxidative stress, chronic inflammation, and low-dose endotoxemia. Senescence encompasses an increase in nuclear p21 and NADPH Oxidase 4 (NOX4) with a concomitant decrease of the antioxidative enzyme Thioredoxin-1 (Trx-1) and endothelial NO Synthase (eNOS). This results in a disturbed redox balance and mitochondrial dysfunction along with increased production of reactive oxygen species (ROS) by these organelles, collectively leading to endothelial dysfunction, which contributes to aging and age-associated cardiovascular diseases.

In an oxidative stress-induced senescence model in primary human endothelial cells, Goy et al. showed that senescent endothelial cells have decreased levels of the antioxidative enzyme Thioredoxin-1 (Trx-1), while the levels of the ROS-producing NADPH Oxidase 4 (NOX4) were elevated [67]. In mice overexpressing NOX4 specifically in the endothelium, in which ROS levels are increased [68], the protein levels of Trx-1 negatively correlated with the expression of NOX4, providing an explanation for the disturbed redox balance in senescent endothelial cells [67] (Figure 3). The importance of redox homeostasis was emphasized by the finding that senescence induction could be inhibited by permanent Trx-1 expression [67]. Induction of endothelial cell senescence with other stimuli was demonstrated by different studies, in which ultrafine carbon nanoparticles [69], low density lipoprotein (LDL) [69,70,71], or concentrations of LPS found in low-dose endotoxemia [63] were used.

Cellular senescence in general is inter alia characterized by the upregulation of senescence-associated beta-Galactosidase, an increase in nuclear p21, and a disturbed redox balance. An additional, specific hallmark of endothelial cell senescence is a decreased production of NO. NO is a key vasodilator that has also been observed to be reduced in endothelial dysfunction [72]. The loss of NO is due to downregulation of endothelial NO Synthase (eNOS), whose levels also decline with age. Moreover, there is less activity of the enzyme, caused by reduced levels of the essential cofactor tetrahydrobiopterin (BH_4_) and the substrate L-arginine [72,73] (Figure 3).

One source of intracellular ROS in senescent cells, among others, is dysfunctional mitochondria [74]. Mitochondrial dysfunction is induced by altered mitochondrial structure due to reduced fusion and fission events, and mitochondrial DNA damage occurs during endothelial cell aging [75]. It is characterized by a reduced respiratory chain capacity per mitochondrion, a reduced mitochondrial membrane potential, and an increased production of ROS. It can lead to senescence and is simultaneously exacerbated by the initiation and maintenance of the senescent phenotype (Figure 3).

## 5. Low-Dose or Metabolic Endotoxemia—Role of the Gut Microbiome and the Endothelium

LPS is present—among other places—in the gastrointestinal tract, where it is ingested through the diet or on the gram-negative bacteria that are naturally present in the gut microbiota. It has been estimated that the human gut is populated with approximately 10^14^ commensal microorganisms, with the majority of them being present in the colon [76]. These include gram-negative bacteria, making them the major source of LPS in the intestinal lumen. Normally, the intestinal epithelium provides an effective barrier against LPS penetration into the bloodstream, such that the median plasma levels are below 15 pg/mL [77,78,79,80]. Consequently, an LPS concentration above 20 pg/mL in the circulation is considered a so-called low-dose endotoxemia, which is set apart from sepsis, where the plasma concentration of LPS is much higher [17]. 

**Figure 4:**
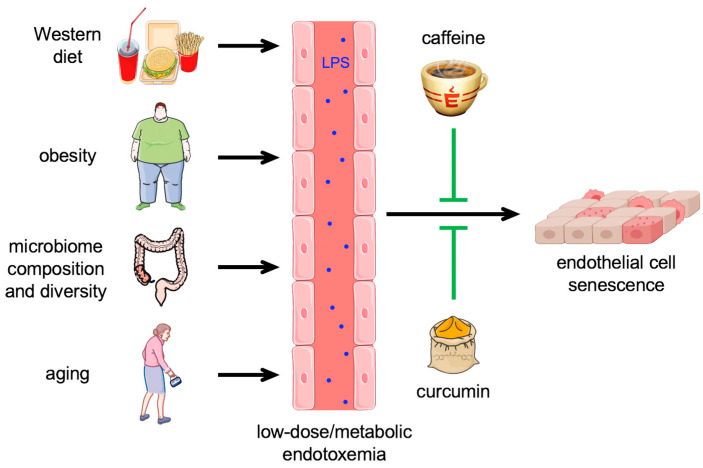
Low-dose endotoxemia-induced endothelial senescence. Under physiological conditions, very small amounts of lipopolysaccharide (LPS) from the intestinal lumen are released into the bloodstream. Various factors, e.g., Western diet, obesity, changes in gut microbiome composition and diversity, and aging, increase the transfer of LPS from the gut to the circulation, resulting in so-called low-dose or metabolic endotoxemia, which leads to endothelial cell senescence. This senescence induction by LPS can be prevented by caffeine and possibly also by curcumin.

Various factors, including an unhealthy diet, obesity, diet-induced changes in microbial diversity, and aging, increase the transfer of LPS from the gut to the bloodstream [81,82,83] (Figure 4). This results in low-dose endotoxemia, which—for the reasons explained below—is also called metabolic endotoxemia.

It has been shown that unbalanced, hyperlipidic meals have a short-term effect on LPS plasma levels, leading to a post-prandial increase already within one hour after a high-fat meal or drink [80,84,85,86]. This rapid increase suggests that dietary lipids facilitate the transfer of LPS from the gut to the bloodstream, and there is evidence that chylomicrons could contribute to the adsorption and transport of LPS [86,87,88].

However, these effects are only transient; an unhealthy diet, on the contrary, leads to a persistent increase in blood LPS levels, which was first demonstrated in rodent models. 4 weeks of a high-fat diet in mice persistently increased the plasma LPS concentration, which usually fluctuates diurnally with the feeding cycles, reaching the highest levels at the end of the feeding period, approximately threefold. As the same effect was obtained by subcutaneous infusion of LPS and the increase in plasma LPS was 10 to 50 times lower than in sepsis, the term metabolic endotoxemia was coined for this phenomenon. In both settings, the increase in circulating LPS was paralleled by the upregulation of inflammatory cytokines, indicating that metabolic endotoxemia entails a low-grade inflammation [89]. An increase in plasma LPS was also observed in mice on high-glucose or high-fructose diets [90] and rats on Western [91] and high-fat/high-sugar diets [92], respectively. Furthermore, metabolic endotoxemia was associated with obesity and insulin resistance [89,90], both being risk factors for the development of cardiovascular diseases. Interestingly, endotoxemia seems to induce these phenomena [89].

Unhealthy diets also induce changes in the gut microbiome [89,90], which seem to be at least in part responsible for the observed metabolic alterations, as dietary interventions that positively affect the composition of the microbiota or treatment with antibiotics can revert some of the effects [93,94]. In that respect, it is noteworthy that not simply the sheer number of gut microorganisms but rather the composition of the microbiome and, thus, the source of LPS is critical for the outcome. While hexa-acylated *E. coli*-derived LPS promotes dysglycemia and inflammation, lower acylated LPS from another bacterial species does not evoke these effects. Moreover, it can even antagonize the dysglycemia caused by *E. coli* LPS in lean mice and improve insulin sensitivity in obese animals [95]. In summary, these data show that diet-induced metabolic endotoxemia is tightly linked to gut microbiome dysbiosis.

Although animal models allow for experimental manipulations not possible in humans, similar correlations and effects have been observed in epidemiological studies and some small-scale clinical trials. A study based on a dietary survey revealed an association between energy intake and endotoxemia in healthy men, and it was suspected that fat is the critical component for the transfer of LPS into the bloodstream [96]. A correlation between dietary habits and metabolic endotoxemia has also been inferred from another study, in which healthy food choices have been associated with lower serum LPS activity [97]. Direct experimental proof for the impact of an unhealthy diet on serum LPS levels was obtained in crossover studies with healthy subjects. Similar to mice, a meal rich in saturated fats induced a post-prandial increase in LPS in the blood [98]. Also, the long-term effects could be recapitulated by demonstrating an increase in plasma endotoxin activity of over 70% after a 4-week Western diet, which returned to baseline after a washout period and another 4 weeks on a prudent diet [99]. Interestingly, experimental endotoxemia in humans induced insulin resistance [100], similar to what has been observed in mice, again underscoring that low-dose endotoxemia leads to metabolic states that increase the risk for cardiovascular diseases.

As in mice, unhealthy diets also have a major impact on the human gut microbiome. Work in humanized gnotobiotic mice, i.e., germ-free animals transplanted with human fecal microbial communities, elegantly showed that a Western diet shifts the gut microbiome already within a day. Interestingly, mice colonized with microbiota from obese Western diet-fed humanized donors developed adiposity [101], similar to what has been described for animals on a 4-week high-fat diet. A correlation between dietary patterns and changes in gut microbiome composition has also been directly demonstrated in humans. Interestingly, controlled consumption of a high-fat/low-fiber or low-fat/high-fiber diet induced alterations already within 24 h, the same time frame as in the gnotobiotic mice [102]. Diet can not only change the ratio between different bacterial species in the gut microbiome, but also has a profound effect on its complexity, which is directly connected to metabolic and inflammatory parameters. A microbiome analysis of nearly 300 non-obese and obese subjects revealed that lower bacterial richness goes along with adiposity, insulin resistance, and more pronounced inflammation [103]. The same observations were made in a parallel study, which additionally demonstrated that weight loss and weight stabilization interventions in obese and overweight individuals can increase microbiome diversity and improve clinical phenotypes [104].

The connection between the intestinal microbiome and chronic diseases or risk factors for their development is also reflected in the elderly. The analysis of fecal microbiota in elderly subjects revealed major changes, which are related to clinical parameters like inflammatory markers and frailty [105,106]. Notably, although a part of these alterations in the microbiome is due to lifestyle, there also seem to be intrinsic changes in the gut microbiota during the aging process, which has been shown in rodent models [107] as well as in humans [108]. An unfavorable composition of the microbiome impairs the barrier function of the gut epithelium, leading to increased transfer of LPS into the bloodstream [109]. Actually, a nearly two-fold increase in plasma LPS levels, and thus metabolic endotoxemia, was demonstrated in aged subjects free of chronic age-related diseases and physical disabilities, when compared to young individuals [29].

As described before, metabolic endotoxemia entails a systemic low-grade inflammation and thereby affects several cell types, also including those in the vasculature, e.g., leukocytes, platelets, and endothelial cells. Therefore, low-grade endotoxemia increases the risk for cardiovascular diseases [79,110]. One of the most critical targets for this metabolic endotoxemia-associated low-grade inflammation is the endothelium, as it plays a central role in vascular homeostasis, such that its dysfunction is associated with nearly all cardiovascular diseases. In fact, a low-grade systemic inflammation triggered by infusion of 2 ng/kg LPS in humans was paralleled by a significant decrease in peripheral endothelial progenitor cells, which are important for the repair of endothelial injuries [111]. However, not only these progenitor cells are affected by metabolic endotoxemia but also mature endothelial cells in the vascular wall. While treatment of primary human endothelial cells with 150 ng/mL of LPS led to activation and apoptosis [16], exposure to 1 ng/mL over a period of 2 weeks induced cellular senescence similarly to treatment with low doses of H_2_O_2_ [63], clearly demonstrating dose-dependent effects of LPS on the endothelium. The induction of endothelial cell senescence by low-dose endotoxemia also explains the association between metabolic endotoxemia and cardiovascular diseases.

Based on the notion that senescence impairs mitochondrial functionality, which is also essential for endothelial cells, it seems attractive to use compounds that positively affect the mitochondria to counteract senescence induction. One such compound is caffeine, a widely consumed drug found in coffee, tea, soft drinks, and chocolate. Large epidemiological studies have demonstrated an association between habitual caffeine intake and a decrease in mortality, including death as consequence of cardiovascular disorders [112,113]. We have previously shown that the consumption of four cups of coffee translates into caffeine serum concentrations of approximately 30 µM [114]. These physiological concentrations improved the migration of endothelial progenitor cells and mature endothelial cells and repair after vascular injury [114]. Moreover, in mice, they improved outcomes after myocardial infarction [115]. This positive effect is due to increased translocation of p27 into the mitochondria and improved electron transport chain activity. Moreover, caffeine reduced age-associated decline in mitochondrial functionality [115].

Another interesting natural compound in this context could be curcumin. This polyphenol is the main active ingredient in the root of *Curcuma longa*, which has been used as a medicinal plant in Asia for over 4000 years. Multiple studies have shown a protective effect of curcumin in cardiovascular diseases (for review, see [116]).

While there is some evidence that curcumin and caffeine play a role in the reduction of the effects of endotoxemia, information on detailed mechanisms underlying these are sparse to date. As these dietary factors have to pass through the digestive tract, one could assume that they interact with and have an impact on the gut microbiome. Some studies hint towards curcumin altering the gut microbiome composition with an increase in beneficial versus pathogenic bacteria (for review, see [117]). Based on the above-discussed impact of the microbiome on plasma LPS levels, it can be speculated that this effect might reduce LPS concentrations in the circulation. Although caffeine can alter the gut microbiome and thereby ameliorate the metabolic syndrome in obese mice [118], this is only very indirect evidence that it might also affect plasma LPS levels, as metabolic syndrome is associated with metabolic endotoxemia.

However, the direct effects of curcumin and caffeine on the endothelium have been investigated in some more detail and will be discussed in the following chapter.

## 6. Impact of Curcumin and Caffeine on High-Dose and Metabolic Endotoxemia with Respect to the Endothelium

As outlined above, upon leakage of the gut, LPS enters the circulation, which in high doses leads to so-called “leaky vessels”, resulting in circulatory and organ failure, finally leading to death within hours or days in mice and humans. The role of curcumin and caffeine treatment in high-dose endotoxemia/sepsis with respect to the endothelium has barely been investigated.

With respect to curcumin, the expression of ICAM-1 was inhibited ex vivo and in vivo in mice, resulting in reduced adhesion of neutrophils and monocytes/macrophages to the endothelium [119]. In rats, curcumin infusion preserved endothelial barrier function in a high-dose LPS model [120]. Of note, these studies only investigated the preventive effects of the compound, as it was administered prior to LPS infusion or treatment. In 2022, the first double-blinded placebo controlled clinical trial with curcumin supplementation in critically ill sepsis patients was performed. Although, the curcumin supplementation was only for 10 days in 20 patients and 20 placebo controls, curcumin significantly improved inflammatory markers, such as Interleukin-1beta, and reduced markers of endothelial activation including ICAM-1. Moreover, it decreased the sepsis-related organ failure assessment score and the duration of mechanical ventilation [121]. Thus, one could assume that curcumin supplementation could improve endothelial functionality in sepsis and may lead to reduced mortality. However, further trials are needed to assess the potential of curcumin with respect to endothelial function and mortality in sepsis patients.

For caffeine, there are currently no studies available that investigate its impact on the endothelium in high-dose endotoxemia/sepsis.

**Figure 5:**
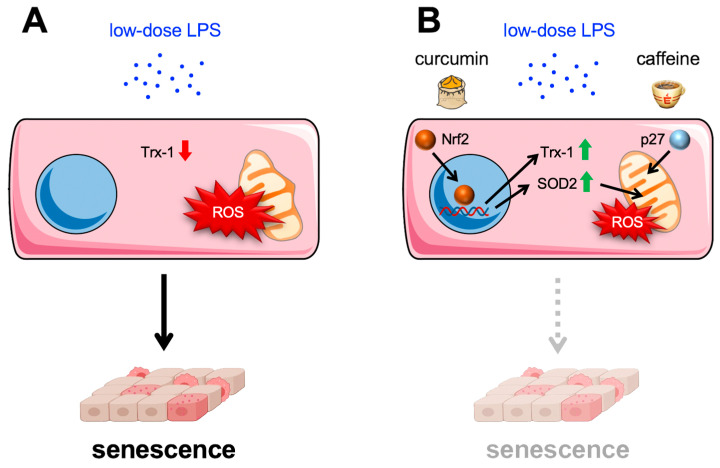
Curcumin and caffeine inhibit low-dose endotoxemia-induced endothelial senescence. (**A**) Low doses of lipopolysaccharide (LPS) in the circulation induce senescence in endothelial cells, which goes along with reduced levels of Thioredoxin-1 (Trx-1), one of the major antioxidative systems, reduced mitochondrial functionality, and increased ROS production. (**B**) Curcumin and caffeine inhibit senescence induction by low-dose endotoxemia through different mechanisms. Curcumin induces stabilization and nuclear translocation, resulting in enhanced expression of Trx-1 and Superoxide Dismutase 2 (SOD2) and reduced ROS production. Caffeine induces the translocation of p27 into the mitochondria, thereby maintaining their functionality.

As described before, low-dose endotoxemia entails endothelial cell senescence. However, the impact of curcumin on metabolic endotoxemia and endothelial cell senescence has not been investigated directly. Nevertheless, there is circumstantial evidence for potential protective effects. Curcumin improved the barrier function in two colon carcinoma cell lines [122], suggesting that it might reduce LPS transfer from the gut to the circulation and thus prevent systemic inflammation, one of the consequences of metabolic endotoxemia. A direct impact of curcumin on inflammation in a disease setting characterized by metabolic endotoxemia has been shown by the reduction of inflammatory cytokine levels in subjects with metabolic syndrome in a randomized controlled trial [123]. In this study, a daily dose of 1 g curcumin was administered over a period of 8 weeks; however, the curcumin concentrations in the blood were not measured. Several studies have measured the serum concentration after administration of crude curcumin ranging from 1 to 3200 ng/mL depending on dose (2 to 12 g) and subject physiology (for review, see [124]). Moreover, short-term treatment of human endothelial cells with curcumin induced stabilization and nuclear translocation of the transcription factor Nrf-2 and enhanced expression of antioxidative defense systems, including Thioredoxin-1 and Superoxide Dismutase 2 [125,126] (Figure 5). This could also be relevant in the context of endothelial cell senescence, which is associated with increased ROS production but has not been studied so far.

In contrast, caffeine effects have been investigated not only in healthy endothelial cells and with respect to vascular repair after injury but also in low-dose endotoxemia-induced endothelial senescence, which was counteracted by caffeine. This was shown by the treatment of endothelial cells with caffeine during senescence induction with low-dose LPS. The co-incubation with both substances blocked the upregulation of nuclear p21, a general senescence marker. Moreover, it also conserved endothelial functionality by maintaining the levels of Trx-1 and eNOS and preserving the migratory capacity of endothelial cells. Mechanistically, this is again due to the translocation of p27 into the mitochondria, as permanent expression of mitochondrially targeted p27 also blocked senescence induction (Figure 5). Interestingly, the same effects were observed when caffeine was applied to already senescent endothelial cells [63]. These data suggest that caffeine can reverse or at least delay endothelial cell senescence induced by metabolic endotoxemia and might, therefore, be an additional protective dietary factor in the context of aging.

This last section highlights that caffeine and curcumin—natural food components—could offer options to combat the negative senescence-inducing effects of low-dose or metabolic endotoxemia.

## 7. Conclusions

In conclusion, dependent on the concentration of LPS in the blood, high-dose or low-dose endotoxemia is induced. High-dose endotoxemia, also called sepsis, often leads to septic shock, endothelial cell death, organ failure, and death. In contrast, low-dose or metabolic endotoxemia leads to chronic inflammation, which results in endothelial cell senescence. Two natural compounds, caffeine and curcumin, seem to inhibit or at least delay low-dose endotoxemia-induced endothelial cell senescence. Thus, natural compounds may have the potential to protect against the negative senescence-inducing effects of metabolic endotoxemia.

## Data Availability

Not applicable.
